# The effect of alcohol advertising, marketing and portrayal on drinking behaviour in young people: systematic review of prospective cohort studies

**DOI:** 10.1186/1471-2458-9-51

**Published:** 2009-02-06

**Authors:** Lesley A Smith, David R Foxcroft

**Affiliations:** 1 School of Health and Social Care, Oxford Brookes University, Oxford, OX3 0FL, UK

## Abstract

**Background:**

The effect of alcohol portrayals and advertising on the drinking behaviour of young people is a matter of much debate. We evaluated the relationship between exposure to alcohol advertising, marketing and portrayal on subsequent drinking behaviour in young people by systematic review of cohort (longitudinal) studies.

**Methods:**

studies were identified in October 2006 by searches of electronic databases, with no date restriction, supplemented with hand searches of reference lists of retrieved articles. Cohort studies that evaluated exposure to advertising or marketing or alcohol portrayals and drinking at baseline and assessed drinking behaviour at follow-up in young people were selected and reviewed.

**Results:**

seven cohort studies that followed up more than 13,000 young people aged 10 to 26 years old were reviewed. The studies evaluated a range of different alcohol advertisement and marketing exposures including print and broadcast media. Two studies measured the hours of TV and music video viewing. All measured drinking behaviour using a variety of outcome measures. Two studies evaluated drinkers and non-drinkers separately. Baseline non-drinkers were significantly more likely to have become a drinker at follow-up with greater exposure to alcohol advertisements. There was little difference in drinking frequency at follow-up in baseline drinkers. In studies that included drinkers and non-drinkers, increased exposure at baseline led to significant increased risk of drinking at follow-up. The strength of the relationship varied between studies but effect sizes were generally modest. All studies controlled for age and gender, however potential confounding factors adjusted for in analyses varied from study to study. Important risk factors such as peer drinking and parental attitudes and behaviour were not adequately accounted for in some studies.

**Conclusion:**

data from prospective cohort studies suggest there is an association between exposure to alcohol advertising or promotional activity and subsequent alcohol consumption in young people. Inferences about the modest effect sizes found are limited by the potential influence of residual or unmeasured confounding.

## Background

The influence of alcohol marketing and advertising on the drinking behaviour of young people is a matter of much debate, mostly focused on the question of whether advertising increases consumption and risky drinking by young people. On the one hand the International Center for Alcohol Policy (ICAP) reported in 2003 to a World Health Organisation (WHO) meeting [1] that there is no compelling evidence of an association between advertising and drinking patterns or rates of abuse among young people, noting that:

*"The industry does not condone promotion and advertising of beverage alcohol to those under the legal minimum purchase age. Yet it should be acknowledged that young people are inevitably exposed to beverage alcohol advertising, as they are to advertising for any other consumer product. They are aware of it, and are able to identify and distinguish between alcohol brands, just as they are able to discern brands of other consumer goods. However, the evidence does not support the notion that such awareness increases consumption by young people." *(point 30, page 9)

On the other hand, healthcare researchers and workers have shown associations between exposure to alcohol advertising and drinking behaviour in cross-sectional surveys [2-5], and it has been argued that an increased awareness of alcohol messages amongst young people might lead to earlier drinking, higher consumption and increased harm, and should be addressed through stronger marketing regulation [6]. Alongside this, macro-level analyses comparing advertising coverage with drinking consumption has been used to provide a rationale for imposing limits on alcohol advertising. One study, drawing on data from Organisation for Economic Co-operation and Development (OECD) countries, reported that total expenditure on alcohol advertising is linked to higher consumption and argued that advertising bans could result in significant reductions in consumption [7]. Similarly, an economic analysis in the United States assessed the effects of alcohol advertising on youth drinking behaviours by comparing federally reported levels of youth drinking with detailed reports on alcohol advertising in local markets during the same years. The analysis concluded that a complete ban on alcohol advertising could reduce monthly levels of youth drinking by 24% and youth binge drinking by about 42% [8]. Correspondingly, in the United States the Institute of Medicine has called for stronger regulation of alcohol marketing [9].

However, causal relationships cannot be directly inferred from these studies and this limits the conclusions that can be drawn about the potential impact of advertising bans. Moreover, the alcohol and advertising industry have used data from econometric studies to argue that advertising bans have little impact on overall alcohol consumption [10-13].

Whether young people are directly targeted by alcohol advertisers or not, they are exposed to alcohol advertising on television, in print media, and on radio. A first question to be answered through rigorous research, therefore, is whether alcohol advertising does have an impact on alcohol consumption amongst young people. This question is best addressed through large prospective cohort studies that examine the relationship between baseline early exposure to alcohol advertising and subsequent consumption and misuse. Helpfully, several such studies have recently been published [14-22].

The aim of our systematic review was to evaluate the likelihood that exposure to alcohol advertising, marketing and portrayal of alcohol increases self-reported alcohol use in young people. We have specifically focused on substantive behavioural outcomes – alcohol use – rather than surrogate outcomes such as brand awareness, or attitudes or intentions towards drinking as the exact causal relationship between surrogate outcomes and subsequent drinking behaviour is unclear. Substantive outcomes provide a more robust basis for evidence based decision making.

Several reviews of the literature on the association of advertising exposure and drinking in young people or, more generally, the effects of media on the behaviour and lifestyles of young people have previously been published [23-31]. However, none use explicit, transparent methodology and they generally lack critical appraisal of individual study weaknesses in relation to any likelihood of bias. These reviews also tend to include weaker study designs, do not clearly distinguish cross-sectional and longitudinal study evidence [4,5,32], focus on clinical/public health aspects rather than methodological detail, and draw major conclusions based on predominantly cross-sectional studies. Our review differs in aim from previous reviews which focused on evaluating the association between media effects and expectancies of drinking or drinking behaviour. Another important difference in our review is the detailed description of our systematic and rigorous approach to the topic, consistent with best methodological practice in systematic reviews of prospective cohort studies, in particular an assessment of the likelihood of bias of reviewed studies [33]. Furthermore, although previous reviews have referenced some of the studies we have included in our review, none have covered all the studies that we have included. Therefore, we provide an update to previous reviews focusing on findings from longitudinal study designs.

## Methods

### Eligibility criteria

We considered studies that evaluated the relationship between alcohol advertising or marketing and alcohol use in young people. We included prospective cohort (longitudinal) studies where young people's exposure to alcohol advertising or attitudes to alcohol advertising and alcohol drinking behaviour were evaluated at baseline and alcohol drinking outcomes were again evaluated after a given period of time. The rationale for restricting the review to prospective cohort studies is that they provide the highest level of evidence that is available for evaluation of advertising and marketing exposure and subsequent drinking behaviour. If such studies are well designed, conducted and analysed they can provide supportive evidence for a causal association between a particular exposure and an outcome. Randomised controlled trials (RCTs), the best design for inferring causality, have not been conducted in this area and are unlikely to be in the future as they are impractical, and it may be unethical to randomise participants or communities to specific advertising and/or marketing strategies in order to evaluate potentially harmful effects.

We excluded experimental studies which evaluated a single exposure to advertising of one form or another and examined immediate effects on either attitude or liking for the advertisements or drinking behaviour. Whilst experimental studies have advantages in that they offer better control over the intervention that participants are exposed to so that the intervention can be more accurately described and causality more confidently inferred; they do not reflect the complexity of the advertising and commercial milieu that people are exposed to in their daily lives, and only evaluate effects post-exposure at a single time-point, so results are not applicable to a broader context. We have also excluded cross-sectional, time-series and econometric studies. Cross-sectional surveys measure the association between a particular exposure such as alcohol advertising and drinking behaviour, but do not show whether the exposure preceded the outcome. Reverse causality cannot be ruled out, whereby young people who drink or misuse alcohol are more receptive to alcohol advertising. Time-series studies are also not ideal for showing temporal relationships due to a greater risk of confounding. One other weakness of the time-series studies is that they measure exposure and outcomes at a population level, rather than in individuals, and therefore include all age groups and are not exclusively focused on young people. Variation in effects in different age groups may be obscured when looking at aggregate population data. Econometric or ecological studies, which may also use time-series data, use data from different sources and statistical modelling to examine relationships between exposure (advertising expenditure) and outcome (alcohol sales). Again these studies are not ideal for this review as they do not specifically look at drinking behaviour in young people but report aggregate alcohol consumption across the population. The observed effect is also highly dependent on the choice and source of factors that are used for the statistical model.

To be included in our review, cohort studies were required: (i) to evaluate young people of school or college age. Studies of participants including young people were excluded if results were not presented separately by age groups or if young people constituted less than 75% of the overall sample; (ii) to evaluate conventional advertising and marketing practices including above and below the line activity, as well as alcohol portrayal in broadcast and print media, for example product placement and depiction of alcohol use. This includes advertising appearing on television, radio, newspapers, billboards, posters, or depiction of alcohol use in movies, TV programmes, music videos and song lyrics, promotional activities including give-aways such as t-shirts and other items bearing alcohol brand logos. Portrayals of alcohol use are particularly prevalent in prime-time programming [34], music videos [35], and during television coverage of sports events [36]; and (iii) to evaluate any alcohol consumption outcome which included: self-reported alcohol use; frequency quantity measures; and self-reported use of specific brands of alcohol or type of alcohol e.g. beer, wine or spirits. We excluded studies reporting only intention to drink as an outcome, or attitude to drinking. Studies only reporting awareness and that did not measure any effects on drinking were also excluded.

### Identification of studies

Electronic databases searched were Medline and Embase from their inception to October 2006. Search terms included free text and MESH terms for drinking behaviour and advertising and marketing. The exact search strategies are shown in Table 1 (see Additional file 1) Reference lists of retrieved reviews and primary studies were also scanned for additional relevant studies. There was no restriction to language of publication.

### Study selection and synthesis

Potentially relevant studies were identified by screening titles and abstracts of retrieved references from the electronic databases. Articles were not selected unless the title or abstract focused on effects of alcohol advertising, marketing or portrayals and on drinking behaviour in young people. Where this was not clear, the full text of the articles was retrieved for further screening. Each retrieved article was screened for review inclusion according to the eligibility criteria described above. Data from included studies were extracted and summarised as a narrative synthesis. Threats to internal and external validity were appraised for each study using the Newcastle-Ottawa Quality Assessment Scale for cohort studies adapted for this review [37]. Quality components assessed were:

#### External validity

1. Was the sample a consecutive sample or a random sample of the population?

2. Did at least 80% of all eligible participants agree to participate?

#### Internal validity

3. Performance bias – was ascertainment of exposure by structured interview?

4 Detection bias – a) was ascertainment of outcome by structured interview? b) Were investigators blind to exposure status or data collected independently?

5. Attrition bias – a) were all participants followed up for the same length of time? b) Were at least 80% of participants included in the final analysis or was the description of those not included unlikely to introduce bias?

6. Control of confounding: a) age or school grade; b) gender; c) ethnicity; d) social influences;

e) social bonds; f) attitudes and behaviour; g) treatment group (participants in an RCT of drug prevention programme); h) TV or other media use; i) parental education; j) school performance; k) self esteem; l) rebelliousness; m) sensation seeking; n) parenting style 0) smoking; p) drinking at baseline q) puberty; r) alcohol sales per capita; s) school status; t) propensity score (accounts for attrition); u) team sport participation; v) = school; w) = living situation; y) = socioeconomic situation.

Studies were awarded an asterix if the component was adequately addressed. For the confounding factors a-y in the selection bias/control of confounding factors section, an asterix indicates that the groups were either balanced or matched for at study start or the variable was adjusted for in an analysis.

Studies not eligible for inclusion were tabulated with reason for exclusion. Screening, selection, data extraction and narrative synthesis were undertaken by one systematic reviewer.

## Results

**Figure 1:**
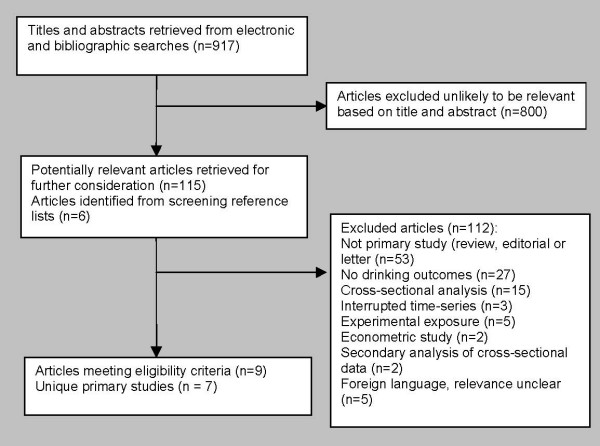
**Results of searches of electronic databases and hand searching**.

The electronic searches identified 915 potentially relevant articles. After screening the titles and abstracts, 115 potentially relevant articles were obtained as full text publications. An additional six articles were identified from screening the reference lists of retrieved articles. After screening each full text article for review eligibility, 112 were excluded leaving nine articles reporting on seven studies for review inclusion, Figure 1. Many studies were excluded mainly because they were secondary reports: reviews, letters or editorials on media effects. We found five foreign language publications without English abstracts requiring translation to determine eligibility but this was beyond the scope of this systematic review. Other articles were excluded mainly due to ineligible study designs: cross-sectional surveys, experimental, time-series or econometric studies. We excluded three articles because although data were taken from a prospective cohort study, these data were from a cross-sectional analysis focusing on just one time point [4,5,38].

### Description of included studies

Nine publications reporting on seven prospective cohort studies were identified that met the review inclusion criteria [14-22]. The seven studies provided data on 13,255 participants aged 10 to 26 years old. Characteristics of the included studies are shown in Table 2 (see Additional file 2). Five were conducted in the USA [16-19,21], one in Belgium [20] and one in New Zealand [14,15,22]. In one study [16] the cohort was part of an RCT of a school-based drug prevention programme, and in another [15] the cohort was a sub-set of a larger cohort study recruited in 1972 and followed through childhood to early adulthood evaluating growth and development.

The age of participants at baseline interview was 12 to 13 years (7^th ^grade) in three studies [15,16,18], 14 to 15 years (9^th ^grade) in one [19], one study [17] recruited a broader age group of youth, 15 to 26 year olds, one [20] used a mixed age group of first (aged 11 to 12 years) and fourth year (aged 14 to 15 years) secondary school students and one [21] used 10 to 14 year olds (5^th ^to 8^th ^grade).

In five studies participants were followed up once after baseline. Time to follow-up was one year [18,20], 18 months [19], 30 months [16] and 13 to 26 months [21]. One study reported outcomes at multiple time-points, six years and nine years and 14 years [14,15,22]. One study evaluated participants at four time points and present results for follow-up after 21 months taking the multiple time points into account in the analysis [17].

Each study used disparate measures of exposure; all relied on self-reported measures. One generated a composite score to reflect the amount of exposure to TV beer advertising, magazine alcohol advertising, beer concession stands and in-store advertising displays [16]. One measured exposure to any alcohol advertising in the past month on each of four media, TV, radio, billboards and magazines [17]. Another classified exposure as watched TV show index to quantify exposure to alcohol ads in specific TV shows in addition to self-reported exposure to alcohol ads [18]. In the study by Connolly [15] recall of alcohol advertisements from different media, TV, radio, magazines, newspapers and films was evaluated. Two studies measured exposure as hours of TV and music video viewing [19,20], and one exposure to alcohol use in popular movies [21].

Drinking status was measured in all studies at follow-up. Two studies reported any alcohol use in the past month [17,18], one study reported alcohol use in the past year [16], one reported frequency of drinking at specific locations and average and maximum amount alcohol consumed on an occasion [15], one reported lifetime and past 30 days alcohol use [19], one alcohol use whilst going out [20], and one incident alcohol use without parental knowledge [21].

### Methodological quality

One study used a random sample of youth [17] three randomly selected schools and all participants at those schools were invited to participate [18,20,21]; in one study [19] all participants at six schools were eligible to participate but how schools were selected was not described; one study used the original sample of participants selected for participation in an RCT but excluded those with missing data [16]; and one study consisted of a sub-sample of children who had exposure and outcome data available at all follow-up periods [15].

Ascertainment of exposure and outcome data were by self-reported questionnaires in four studies [16,18-20], by face-to-face interview in one [15] and computer-aided telephone interview in two [17,21]. None of the studies explicitly reported that interviewers were unaware of the exposure status of participants when outcome assessments were conducted, however with participants independently reporting drinking outcomes via self-reported questionnaires there is little scope for detection bias on the part of the investigators. Not all children were non-drinkers at baseline. Two studies reported results for baseline drinkers and non-drinkers separately [16,19].

All studies suffered, to a greater or lesser extent, from potential attrition bias. Attrition rates were 33% [21] and 69% [17] in two surveys where data were collected by telephone; 18% [16], 25% [18], 39% [19] and 36% [20] in surveys conducted in schools, and 35% [15] for the survey with face-to-face interviews and questionnaires.

One study used imputation to account for missing data [16]; all other studies excluded participants with missing data from the analyses.

Statistical adjustments for measured confounding factors were performed by each study, but the number and type of confounders varied from study to study. The results of the overall quality assessment of each study are shown in Table 3 (Additional file 3).

### Study findings

Connolly [15] investigated the relationship between alcohol consumption at 18 and alcohol-related mass media communications recalled at ages 13 and 15 years in a New Zealand cohort of young people. Among men, those who recalled more alcohol advertisements at age 15 drank significantly more beer at 18 years (average amount of beer consumption p = 0.047; maximum amount of beer consumption p = 0.008). In women a negative association of alcohol advertisement recall at age 13 years and frequency of drinking beer was found (p = 0.029). Multi-variate analyses were adjusted for potential confounders which were: media exposure, gender, current occupation, living situation, socio-economic status and peer approval of drinking. There was no significant effect on wine or spirit consumption in either women or men. Whilst significant relationships were detected, we cannot rule out the possibility they occurred due to chance. The authors reported results for more than 35 statistical tests and significant findings would be expected to occur due to chance. This coupled with the small sample sizes, 251 men and 184 women, cast some doubt on these findings being true effects. Longer follow-up from this same sample at age 21 and 26 years have been published [14,22]. In the group that were beer drinkers at 18 years, liking of alcohol advertising and brand allegiance had a positive impact on beer consumed at age 21 years; standardised coefficients were 0.26 and 0.36, respectively. At 26 years, those showing a liking for alcohol advertising at 18 years were more likely to be in a group of heavier drinkers.

Stacy [18] assessed the impact of exposure to TV alcohol advertisements on alcohol use in 2,250 12 to 13 years old school children in California followed up for a year. At baseline, 16% reported drinking beer in the past month, 15% reported drinking wine in the past month, and 8% reported three-drink episodes in the past month. At follow-up, prevalence was 18% for beer, 20% for wine and 12% for three-drink episodes. At one-year follow-up, each standard deviation increase in TV viewing of programmes with alcohol advertisements at baseline was associated with a significant increase (44%) in risk of beer use ((odds ratio (OR) 1.44 95% Confidence Interval (CI): 1.27 to 1.61)), wine/liquor use (OR 1.34; 95% CI: 1.17 to 1.52) and three-drink episodes (OR 1.26; 95% CI: 1.08 to 1.48), controlling for general TV viewing frequency, participation in team sports, perception of peer alcohol use, perceived peer approval of alcohol use, intentions to use alcohol, perceptions of adults alcohol use, gender, ethnicity and school, exposure memory covariates and a propensity score to adjust for differential risk profile of those lost to attrition. A watched TV sports index was only positively associated with beer drinking, (OR 1.20; 95% CI: 1.05 to 1.37) with adjustment for confounders, and self-reported frequency of exposure was significantly associated with increased risk of beer drinking, (OR 1.21; 95% CI: 1.14 to 1.41). Other exposure measures, cued-recall memory test and draw-an-event memory test, did not show significant relationships with any of the outcomes, though most showed effects in the direction of positive associations with one exception, participants scoring one standard deviation above the mean for draw-an-event memory test were significantly less likely to drink beer one year later (OR 1.14; 95% CI: 1.01 to 1.25).

Ellickson [16] examined the relationship between a range of advertisement exposures and subsequent drinking among US adolescents age 12 to 13 years. Forty-eight per cent non-drinkers at baseline (n = 1,905) initiated drinking by two-year follow-up. For baseline non-drinkers, exposure to in-store beer displays predicted drinking onset at follow-up, OR 1.42 (p < 0.05) adjusted for general TV viewing, social influences, social bonds, gender, ethnicity and attitudes and behaviour. Exposure to TV beer advertisements, magazines with alcohol advertisements, and in-store advertisement displays all showed positive associations, though none were significant in adjusted analyses, OR 1.05, 1.12 and 1.06, respectively. Confidence intervals were not reported for any of the ORs. Among baseline drinkers (n = 1,206), 77% reported alcohol use in the past year at follow-up. Exposure to magazines with alcohol advertisements and to beer concession stands at sports or music events predicted frequency of drinking at follow-up, regression coefficient 0.10 and 0.09, (p-value < 0.05), respectively. Exposure to TV beer advertising or in-store advertisement displays were not significant predictors of drinking frequency in analyses adjusted for baseline drinking and multiple control variables regression coefficient -0.01 and 0.02, respectively.

Snyder [17] evaluated the relationship between self-reported advertising exposure to four media (TV, radio, billboards and magazines) and the prevalence of advertising in the same media sources and alcohol consumption in 15 to 26 year olds in 24 media markets in USA. Participants were followed up at four time-points over a 21 month period. Sixty-one per cent had at least one drink in the past month at baseline and consumed an average of 38.5 drinks a month. Participants reported seeing an average of 22.7 alcohol advertisements per month. For each additional advertisement seen, the number of drinks consumed increased by 1% Event Rate Ratio (ERR) 1.01 (95% CI: 1.01 to 1.02). Also for each additional dollar per capita spent on advertising the number of alcoholic drinks consumed per month increased by 3% ERR 1.03 (95% CI: 1.01 to 1.05). In the sub-group of participants aged less than 21 years (60% of sample), who were below the legal drinking age, similar patterns were seen, ERR 1.01 (95% CI: 1.0 to 1.02) and 1.03 (95% CI: 1.0 to 1.06) increase in number of drinks consumed per month for self-reported advertising exposure and advertising expenditure, respectively. All analyses were adjusted for gender, age, ethnicity, school status and alcohol sales per capita, however the high degree of attrition in this study (more than 50% for two of the four follow-up assessments) precludes firm conclusions on the basis of these findings.

Two studies evaluated exposure to TV and music videos and alcohol use in adolescents [19,20]. In the study by Robinson et al[19] the association between hours of TV, music video and videotape viewing, computer and video game use and subsequent alcohol use at 18 months follow-up was investigated in 1,533 14 to 15 year olds from six public high schools in California. During follow-up, 325 (36.2%) baseline non-drinkers began drinking and 322 (50.7%) drinkers continued to drink. In baseline non-drinkers (n = 898), onset of drinking was significantly associated with hours of TV viewing at baseline. For each additional hour of TV viewing per day the average increased risk of starting to drink during the next 18 months was 9% OR 1.09 (95% CI: 1.01 to 1.18), for each additional hour of music video viewing OR 1.31 (95% CI: 1.17 to 1.47). For each additional hour of videotape viewing the average risk decreased, 11% OR 0.89 (95% CI: 0.79 to 0.99) in analyses controlling for age, sex, ethnicity and other media use. Computer and video game use was not significantly associated with subsequent onset of drinking, OR 0.94 (95% CI: 0.84 to 1.05). In baseline drinkers (n = 635), there were no significant associations between baseline media use and maintenance of drinking. For each additional viewing hour per day the risk, OR (95% CI), of maintenance of drinking was: 1.01 (0.93, 1.11) for television, 1.05 (0.95, 1.17) for music videos, 0.97 (0.86, 1.10) for videos and 1.00 (0.89, 1.12) for computer or video games.

Van Den Bulck [20] examined the relationship between television viewing and music video exposure and subsequent alcohol consumption while going out one year later in 2,546 first and fourth year secondary school students in Flanders, Belgium. Only 65% of the original sample with complete data at both time-points was analysed. The majority of students (63.6%) watched music videos at least several times a week, about a third watched daily. Overall television viewing and music video viewing at baseline significantly predicted the amount of alcoholic beverages adolescents consumed while going out at follow-up. Results of a regression model controlling for gender, school year, smoking and pubertal status were reported: R^2 ^= 0.568 (F = 230.374; df = 7; p < 0.0001).

Sargent [21]evaluated the exposure to alcohol use in popular contemporary movies in a cross-sectional survey with prospective follow-up of never drinkers and recorded incident alcohol drinking 13 to 26 months later. Adolescents, 10 to 14 years old, were recruited from 15 randomly selected schools in New Hampshire and Vermont, USA. Never-drinkers at baseline were followed up (n = 2,406). Baseline median exposure to alcohol use in 601 movies was 8.6 hours, (inter-quartile range (IQR): 4.6 to 13.5). At follow-up, 14.8% reported having tried alcohol, which was significantly associated with alcohol exposure (viewing hours). For each additional hour of movie alcohol exposure the risk of initiating alcohol use was increased by 15%, OR 1.15 (95% CI: 1.06, 1.25) adjusted for school grade, school, gender, parent education, sensation seeking, rebelliousness, self-esteem, school performance, parenting style and smoking experimentation.

## Discussion and conclusion

This systematic review of seven cohort studies on over 13,000 participants shows some evidence for an association between prior alcohol advertising and marketing exposure and subsequent alcohol drinking behaviour in young people. All seven studies demonstrated significant effects across a range of different exposure variables and outcome measures. These included exposure to direct advertising using broadcast and print media and indirect methods such as in-store promotions and portrayal of alcohol drinking in films, music videos and TV programmes. The consistency of effect across a heterogeneous group of studies may be considered a strength.

Notably, three studies showed that onset of drinking in adolescent non-drinkers at baseline were significantly associated with exposure. Robinson [19] showed that for each additional hour of TV viewing per day the risk of starting to drink increased by 9% during the following 18 months. Sargent [21] found that for additional hour of exposure to alcohol use depicted in popular movies there was a 15% increase in likelihood in having tried alcohol 13 to 26 months later. Ellickson [16] showed that exposure to in-store beer displays significantly predicted drinking onset two years later. Effects were less clear in baseline drinkers, whilst greater exposure predicted greater drinking frequency, analyses adjusting for possible confounding factors failed to detect significant relationships.

In studies on mixed groups of drinkers and non-drinkers, increased frequency of TV viewing and music video viewing was highly significantly related to the amount of alcohol consumed while going out [20]. In the study by Snyder [17] of US individuals aged 15 to 26 years, for each additional advertisement seen the number of drinks consumed increased by 1%.

Of interest, to our knowledge, at least two more prospective cohort studies meeting our inclusion criteria have been published since our review was completed [39,40]. Since updating our searches for all new studies is beyond the original scope of the project, we have not incorporated these two studies into the main body of the review. Nevertheless, it is important to note that both of these studies also showed significant relationships between receptivity to alcohol marketing or alcohol advertising in young people. Eleven year olds in the highest centile of exposure to TV beer advertisements, alcohol ads in magazines, in-store beer displays and beer concessions, radio listening time and ownership of beer promotional items were 50% more likely to be drinkers than youth in the lowest centile of exposure one year later controlling for demographic and psychosocial factors and prior drinking [39]. In a sample of non-drinkers aged 11 to 15 years, those reporting high receptivity to alcohol marketing defined as owning or wanting to own alcohol branded promotional items were 77% more likely to initiate alcohol use one year later compared with youth reporting minimal receptivity adjusted for demographic and psychosocial factors and social influences to drink [40].

There are several limitations that should be considered when interpreting the results of this review. Whilst we made an *a priori *decision to only include and review cohort studies which potentially are less likely to suffer from systematic bias than less robust study designs such as cross-sectional surveys or interrupted time series studies, it is nonetheless important to note that cohort studies are also susceptible to bias if not designed and executed using rigorous standards. One of the biggest threats to the validity of observational studies such as cohort studies is the issue of confounding, whereby the outcome of interest is influenced by some other factor or factors in addition to the exposure of interest. Whereas all of the studies controlled for a variety of confounding factors possibly related to alcohol drinking behaviour, unmeasured or unknown confounders cannot be adjusted for and it is not possible to know if residual confounding influenced the analysis. For example, alcohol expectancies, family history, peer influence and personality characteristics may act as confounders in the relationship between exposure to advertising and marketing and subsequent alcohol use. Given the magnitude of the effect sizes shown in these studies, we cannot rule out the possibility that they were due to the effects of residual and unmeasured confounding [41]. However, previous work evaluating smoking exposure in movies and smoking behaviour in adolescents using a simulation model showed that effects of unknown or unmeasured confounders would need to be large in order to overturn the results [42]. Given that no observational study can control for all unmeasured or unknown confounders, researchers may wish to consider using similar approaches to determine the potential impact of such confounders.

Whilst these studies suggest that exposure to advertising and alcohol portrayal in the media increase likelihood of later alcohol consumption, they are unable to inform us how exposure brings about these changes, or what aspects of advertising and marketing are the active components. The extent to which psychological factors determine subsequent behaviours is a worthwhile topic for further study. One study [43] has examined how persuasive alcohol media messages were associated with concurring beliefs and behaviours among youth, concluding that existing exposure based studies do not adequately account for the complex psychological causal mechanisms that may moderate or mediate the relationship between exposure and outcome. However, this analysis is based on cross-sectional data; further studies with longitudinal analyses are desirable. If a better understanding of the relationship of the intermediate steps between exposure and subsequent behaviours can be obtained, then our understanding of the mechanisms of action of alcohol advertising and marketing would be improved. This question, together with lessons learned from the collective experiences of conducting cohort studies [44], should inform the design of future cohort studies.

One other serious threat to the validity of these studies was the degree of attrition in some of the studies. Losses to follow-up between assembly of the cohort and follow-up are inevitable but the aim is to keep this to a minimum as attrition bias may be introduced if reasons for missing data or loss to follow-up are related to exposure or outcome. If adolescents who were lost to follow up were more likely to be drinkers, or at high risk of drinking as found in three of the studies [17,19,21], then this may then lead to underestimating the relationship between advertising and drinking. Generalisability of the results is also affected if losses are in one specific subgroup of participants, and the subsequent loss of power is also a problem with attrition. Of note, none of the studies reported how they estimated sample sizes required. In general, assessment of the design and conduct of the cohort studies reviewed was hampered by the lack of important methodological detail, and fell short of the current recommendations as set out in the STROBE statement [45].

We cannot rule out the possibility of publication bias, whereby studies failing to detect significant relationships were not published, or studies for which selective reporting of only positive associations were published. Of course it is also possible that studies showing positive associations, if sponsored by the alcohol industry or other commercial organisations with a vested interest in advertising or marketing of alcohol, have not been published. Therefore, it is not possible to predict the likely impact of unpublished data on the results of this review. It is also possible that published studies were not found by our search as a fully comprehensive search of databases other than Medline and Embase and other sources only covering the social science literature was not possible within the scope of the limited funding for this review. Attempts, however, were made to locate all available studies by supplementing searches of databases with hand searching reference lists of key reviews and primary studies, which identified many articles published in journals not covered by Medline and Embase.

The results of these cohort studies are supported by findings in cross-sectional surveys which consistently report associations between increased exposure to alcohol advertising or marketing and drinking behaviour [2-5], intentions to drink [46] or advertising awareness and liking [2,47-49]. Although, in one interrupted time-series study countries with advertising bans had lower levels of alcohol consumption and road traffic fatalities [50], others failed to demonstrate significant effects [51,52]. The rationale for the exclusion of these studies is outlined in the methods, and their exclusion would only be a concern if they generally showed a strong effect in the opposite direction.

One question that remains is whether early drinking behaviour shown in these cohort studies is predictive of risky or harmful drinking or alcohol-related problems in the future. Drinking onset at an earlier age has been shown to be associated with a greater likelihood of alcohol dependence in several cross-sectional studies [53-55]. More recently, prospective cohort studies have also shown clear and significant associations between age of onset of drinking and subsequent heavy drinking and alcohol-related problems [56-59].

Given the large budgets allocated to advertising and promotional activity by the alcohol industry, a paucity of research exists evaluating the effects of this advertising. Further research exploring the potential causal impact is warranted; the role of mass media as a potential source of influence on alcohol related knowledge and behaviour of young people has been neglected in many countries [60].

The data from these studies suggest that exposure to alcohol advertising in young people influences their subsequent drinking behaviour. The effect was consistent across studies, a temporal relationship between exposure and drinking initiation was shown, and a dose response between amount of exposure and frequency of drinking was clearly demonstrated in three studies [17,20,21]. It is certainly plausible that advertising would have an effect on youth consumer behaviour, as has been shown for tobacco [61] and food marketing [62].

Does this systematic review provide evidence that limiting alcohol advertising will have an impact on alcohol consumption amongst young people? Not directly: as we noted earlier we can not rule out that the effects demonstrated in these studies is due to residual confounding. Counter-advertising [30], social marketing techniques [63] or other prevention options such as parenting programmes [64], price increases and limiting availability may offer more potential to limit alcohol problems in young people. Nonetheless, we now have stronger empirical evidence to inform the policy debate on the impact of alcohol advertising on young people, and policy groups may wish to revise or strengthen their policy recommendations in the light of this stronger evidence [1,9].

## Competing interests

Foxcroft has received funding from Diageo for a project to develop and evaluate a family-based prevention programme. Smith has not directly received funding from the alcohol industry although has benefited indirectly from the funding to Foxcroft.

## Authors' contributions

DF helped frame the research question and scope of the systematic review. LS developed the protocol, undertook searching, study appraisal, data extraction and synthesis. LS drafted the paper and DF contributed additional material to the Introduction and Discussion sections. Both authors act as guarantors.

## Pre-publication history

The pre-publication history for this paper can be accessed here:



## Supplementary Material

Additional file 1 **Table 1.** Systematic review search strategies. Click here for file

Additional file 2 **Table 2.** Characteristics of prospective cohort studies included in the systematic review. Click here for file

Additional file 3 **Table 3.** Assessment of likelihood of bias of included prospective cohort studies. Click here for file
