# Supplementary material for: The effect of alcohol advertising, marketing and portrayal on drinking behaviour in young people: systematic review of prospective cohort studies
Source: BMC Public Health. 2009 Feb 6;9:51. doi: 10.1186/1471-2458-9-51 (PMC2653035; doi:10.1186/1471-2458-9-51)
Supplement: Additional file 1 — Table 1. Systematic review search strategies. [file 1471-2458-9-51-S1.doc]

Table 1: Systematic review search strategies

Medline (OVID) search strategy

| 1 | Drinking behaviour. Explode all fields |
| --- | --- |
| 2 | Alcohol drinking. Explode all fields |
| 3 | Alcohol* OR drink*.ti.ab.mh |
| 4 | Alcohol related disorders. Explode all fields |
| 5 | 1 OR 2 OR 3 OR 4 |
| 6 | Marketing OR advertising. Explode all fields |
| 7 | 5 AND 6 |

Embase (OVID) search strategy

| 1 | Drinking behaviour. Explode all fields |
| --- | --- |
| 2 | Alcohol drinking. Explode all fields |
| 3 | Alcohol* OR drink*.ti.ab.mh |
| 4 | Abuse OR addiction OR alcoholism. Explode all fields |
| 5 | Alcohol related disorders |
| 6 | 1 OR 2 OR 3 OR 4 OR 5 |
| 7 | Marketing OR advertising. Explode all fields |
| 8 | 6 AND 7 |
