# Supplementary material for: The effect of alcohol advertising, marketing and portrayal on drinking behaviour in young people: systematic review of prospective cohort studies
Source: BMC Public Health. 2009 Feb 6;9:51. doi: 10.1186/1471-2458-9-51 (PMC2653035; doi:10.1186/1471-2458-9-51)
Supplement: Additional file 2 — Table 2. Characteristics of prospective cohort studies included in the systematic review. [file 1471-2458-9-51-S2.doc]

Table 2: Characteristics of prospective cohort studies included in the systematic review

| **Study** | **Participants** | **Exposure** | **Outcomes** |
| --- | --- | --- | --- |
| Study: Connolly [15]  Baseline survey: 1985-86  Follow-up: Six years  Location: New Zealand | Sample: n=667, sub-set of Dunedin cohort study of children recruited in 1972, participants are those who were present for alcohol interviews at age 13, 15 and 18 years. 65% followed up  Sex: n=251 men, n=184 women  Ethnicity: not stated  Data collection: Face-to-face interviews age 13 and 15, self-completed questionnaire age 18 years | Number of advertisements recalled. Mainly included product advertising and sponsorship by alcohol companies, number of moderation messages recalled, number of alcohol portrayals recalled from entertainment material. Type of media was asked about separately: TV, radio, magazines, newspapers and films | Alcohol consumption: total frequency of drinking at a list of drinking locations, average amount alcohol consumed on an occasion, maximum amount consumed on an occasion, |
| Study: Casswell [14]  Baseline survey: 1990/91  Follow-up: Three years  Location: New Zealand | Sample: n=630, same sample as above, participants are those who were beer drinkers at age 18 (all analysed)  Ethnicity: largely of British ancestry  Data collection: computer-based questionnaire and face-to-face supplementary interview by experienced interviewers | Recall of liking of alcohol advertising; brand allegiance assessed by identifying favourite brand of beer | Annual consumption of beer calculated from frequency and quantity at five popular locations (own home, someone else’s home, hotel, tavern or bar, sport’s clubs and night clubs).  Self-reported aggression related to drinking |
| Study: Casswell [22]  Baseline survey: 1990/91  Follow-up: Eight years  Location: New Zealand | Sample: n=799, same sample as above , participants are those who participated in alcohol assessments at age 18, 21 and 26, n=714 (89.4%) analysed (drinkers at each assessment)  Ethnicity: largely of British ancestry  Data collection: computer-based questionnaire and face-to-face supplementary  interview by experienced interviewers | Liking of adverts related to alcohol at age 18 years (baseline) | Yearly frequency and typical quantity of alcohol consumed at five popular locations (own home, someone else’s home, hotel, tavern or bar, sport’s clubs and night clubs).  Self-reported aggression related to drinking |
| Study: Stacy [18]  Baseline survey: Spring 2000  Follow-up: one year  Location: LA, USA | Sample: 2998 seventh grade students in 20 middle schools, schools randomly selected, 2250 (75%) analysed at follow-up  Sex: 51% female  Ethnicity: Hispanic 55%, Asian 19%, non-Hispanic white 14%, African-American 2%, pacific islander 1%, native American 1%, multi-ethnic 5%, 3% did not report  Data collection: self-completed questionnaire administered in class | Watched TV shows index: frequency of specific TV shows viewed and alcohol ad frequency; watched TV sports index created by viewing frequency weighted by average monthly alcohol advertising frequencies; self-reported frequency exposure to alcohol ads rated on a 7-point Likert-type scale; cued recall memory test index calculated as number of recent beer advertisements correctly identified as beer advertisements; event memory test score was number of alcohol advertisements a student could recall across 3 tests | Current alcohol use defined as frequency of at least one drink of beer, wine, wine coolers or liquor in last 30 days; prior use frequency of alcohol in last 6 months and lifetime |
| Study: Ellickson [16]  Baseline survey: Autumn 1997  Follow-up: Spring 2000  Location: USA | Sample: 3,111 children in 7^th^ grade, 82.3% of original sample, excludes participants with missing data  Sex: 50% female  Ethnicity: 88% white, 6.3% Native American, 5.4% other  Data collection: Self-completed questionnaires administered during school hours supervised by trained staff, make-up sessions and mailed surveys for absentees | Television beer advertising, magazines with alcohol advertisements, beer concession stands, in-store advertisement displays; frequency of advertisement used to generate an overall score to give measure of exposure for each of the 4 types | Alcohol use in the past year (none, one to two times, three to ten times, 11-20 times, more than 20 times) |
| Study: Snyder [17]  Baseline survey: April to July 1999  Follow-up: 21 months  Location: USA | Sample: Random sample youth aged 15 to 26 years from 24 media markets, mean response rates across markets 27%, N=1872, 1173, 787 and 588 (31%) at each time point.  Sex: 49% female  Ethnicity: white=70%, black 11.4%, Hispanic 8.2%  Data collection: computer-aided telephone interviewing at four time points during follow-up | 1.Self-reported beer or liquor and pre-mixed drink advertising in past month on each of 4 media (TV, radio, billboards and magazines) summed to produce an overall index score  2. Market level measures of alcohol advertising expenditure-per-capita index calculated from amount spent on alcohol advertisements on TV, radio, newspapers and billboards in each market in 1999 and 2000. | Any alcoholic beverage drinking frequency past four weeks; average quantity alcohol drank; maximum quantity on any occasion. Alcohol use then quantified as number of alcoholic drinks consumed in last month |
| Study: Robinson [19]  Baseline survey: October/November 1994  Follow-up: 18 months  Location: USA | Sample: n=2,609 (94%) of eligible students participated, 1,583 (61%) assessed at follow-up, of which 1,533 analysed. 9^th^ grade students from 6 public high schools, all students eligible if no special needs and proficient in English language  Sex: 50.6% female  Ethnicity: Latino 34%, Asian 34%, White 19%, African-American 5%, other 8%  Data collection: Pencil and paper self-assessments supervised by trained staff | Weekly total hours of TV, music video and videotape viewing, computer and video game use | Lifetime and past 30 day alcohol use. Lifetime drinking defined as a part of one drink. New onset defined as onset of lifetime drinking among baseline non-drinkers. Maintenance of drinking at follow-up, in baseline drinkers, defined as increase in lifetime drinking plus drinking at least once in past month |
| Study: Van Den Bulck [20]  Baseline survey: February 2003  Follow-up: 1 year  Location: Belgium | Sample: n=3022 students eligible, one school excluded due to prior knowledge about study purpose, 2,546 in final sample, 1,648 (64%) Students analysed with complete data at both time-points, all 1^st^ and 4^th^ year students at 15 randomly selected secondary schools  Sex: 45.4% female  Ethnicity: Flemish  Age: 52% 1^st^ year, 48% 4^th^ year students  Data collection: Self-administered questionnaire | Frequency of music video watching, total TV viewing hours per day | Quantity of alcohol consumption while going out (bar, party or disco etc) |
| Study: Sargent [21]  Baseline survey: September 1999  Follow-up: average 17 months (range 12 to 26)  Location: USA | Sample: n=3,577 students who had never tried drinking, 2,406 (67.3%) analysed at follow-up all 5^th^ - 8^th^ grade students at 15 randomly selected middle schools  Sex: about 50% female  Ethnicity: Mostly white  Age: 10 to 14 years  Data collection: Computer-assisted telephone interview | Exposure to movie alcohol use in 50 popular contemporary films, summarized as a score | Ever had beer, wine or other drink with alcohol that your parents didn’t know about? |
