# Supplementary material for: The effect of alcohol advertising, marketing and portrayal on drinking behaviour in young people: systematic review of prospective cohort studies
Source: BMC Public Health. 2009 Feb 6;9:51. doi: 10.1186/1471-2458-9-51 (PMC2653035; doi:10.1186/1471-2458-9-51)
Supplement: Additional file 3 — Table 3. Assessment of likelihood of bias of included prospective cohort studies. [file 1471-2458-9-51-S3.doc]

Table 3: Assessment of likelihood of bias of included prospective cohort studies

| Study | External validity | | Internal validity | | | | | | | | | | | | | | | | | | | | | | | | | | | | | |
| --- | --- | --- | --- | --- | --- | --- | --- | --- | --- | --- | --- | --- | --- | --- | --- | --- | --- | --- | --- | --- | --- | --- | --- | --- | --- | --- | --- | --- | --- | --- | --- | --- |
|  |  |  | Perf bias | Detection bias | | Attrition bias | | Selection bias/control of confounding | | | | | | | | | | | | | | | | | | | | | | | | |
|  | 1 | 2 | 3 | 4a | 4b | 5a | 5b | 6a | b | c | d | e | f | g | h | i | j | k | l | m | n | o | p | q | r | s | t | u | v | w | x | y |
| Ellickson 2005 |  | * |  |  | * | * | * | * | * | * | * | * | * | * | * |  |  |  |  |  |  |  | * |  |  |  |  |  |  |  |  |  |
| Connolly 1994 | * |  | * |  | * | * |  | * | * |  | * |  |  |  | * |  |  |  |  |  |  |  |  |  |  |  |  |  |  | * | * | * |
| Sargent 2006 | * | * | * | * | * | * |  | * | * |  |  |  |  |  |  | * | * | * | * | * | * | * | * |  |  |  |  |  | * |  |  |  |
| Robinson 1998 | * | * |  |  | * | * |  | * | * | * |  |  |  |  | * |  |  |  |  |  |  |  | * |  |  |  |  |  |  |  |  |  |
| Van Den Bulck 2005 | * | * |  |  | * | * |  | * | * |  |  |  |  |  |  |  |  |  |  |  |  | * |  | * |  |  |  |  |  |  |  |  |
| Snyder 2006 | * | * | *§ | * |  | * |  | * | * | * |  |  |  |  |  |  |  |  |  |  |  |  |  |  | * | * |  |  |  |  |  |  |
| Stacy 2004 | * | * |  |  | * | * |  | * | * | * | * | * | * |  | * |  |  |  |  |  |  |  |  |  |  |  | * | * | * |  |  |  |

An asterix (*) indicates that the component was adequately addressed in the study. For the confounding factors a-y in section 6, an asterix indicates that the variable was either balanced or matched for at study start or adjusted for in the analysis. § = expenditure-per-capita obtained from media sales data

External validity: the extent to which the results of the study apply to other situations.

1: Sample a consecutive or random sample; 2: at least 80% of eligible participants recruited

Internal validity: the extent to which bias is minimised in a study.

3: Performance bias – ascertainment of exposure by structured interview; 4: detection bias – a) ascertainment of outcome by structured interview; b) assessors of drinking status blind to exposure status or data collected independently. 5: Attrition bias – systematic differences in follow-up; a) all groups followed up for the same length of time; b) complete follow-up of all participants or at least 80% of sample

6: Selection bias/control of confounding

a = age/grade; b = gender; c = ethnicity; d = social influences; e = social bonds; f = attitudes and behaviour; g = treatment group; h = tv viewing or other media use; i = parental education; j = school performance; k = self esteem; l = rebelliousness; m = sensation seeking; n = parenting style, o = smoking; p = drinking at baseline; q = puberty; r = alcohol sales per capita; s = school status (high school, college, not in school) t = propensity score; u = team sport participation ; v = school; w = living situation; y = socioeconomic status
